# Assessment of gait and posture characteristics using a smartphone wearable system for persons with osteoporosis with and without falls

**DOI:** 10.1038/s41598-023-27788-w

**Published:** 2023-01-11

**Authors:** Krupa B. Doshi, Seong Hyun Moon, Michael D. Whitaker, Thurmon E. Lockhart

**Affiliations:** 1 Division of Endocrinology, Mayo Clinic, Scottsdale, AZ 85259 USA; 2 School of Biological and Health Systems Engineering, Arizona State University, Tempe, AZ 85281 USA

**Keywords:** Biomedical engineering, Bone, Risk factors

## Abstract

We used smartphone technology to differentiate the gait characteristics of older adults with osteoporosis with falls from those without falls. We assessed gait mannerism and obtained activities of daily living (ADLs) with wearable sensor systems (smartphones and inertial measurement units [IMUs]) to identify fall-risk characteristics. We recruited 49 persons with osteoporosis: 14 who had a fall within a year before recruitment and 35 without falls. IMU sensor signals were sampled at 50 Hz using a customized smartphone app (Lockhart Monitor) attached at the pelvic region. Longitudinal data was collected using MoveMonitor+ (DynaPort) IMU over three consecutive days. Given the close association between serum calcium, albumin, PTH, Vitamin D, and musculoskeletal health, we compared these markers in individuals with history of falls as compared to nonfallers. For the biochemical parameters fall group had significantly lower calcium (*P* = 0.01*) and albumin (*P* = 0.05*) and higher parathyroid hormone levels (*P* = 0.002**) than nonfall group. In addition, persons with falls had higher sway area (*P* = 0.031*), lower dynamic stability (*P* < 0.001***), gait velocity (*P* = 0.012*), and were less able to perform ADLs (*P* = 0.002**). Thus, persons with osteoporosis with a history of falls can be differentiated by using dynamic real-time measurements that can be easily captured by a smartphone app, thus avoiding traditional postural sway and gait measures that require individuals to be tested in a laboratory setting.

## Introduction

Injuries associated with falls continue to pose a substantial burden for older adults both in human suffering and economic losses. Falls among older adults are also a growing public health concern and are responsible for over 684,000 deaths and nearly 37.3 million annual visits for medical intervention worldwide^1^. In the Unites States of America, costs for fatal and nonfatal fall-related injuries in 2015 were approximately $50 billion, and medical expenditures for fatal falls were estimated at $754 million^2^. Of 2.4 million emergency department visits in 2018 among adults aged 65 years and older, unintentional falls were responsible for approximately 90% of injury-related visits^3^. Falls are also the most common reason for older persons being forced to transition from independent living to assisted care^4,5^. With this transition often comes a decrease in quality of life^6^ and a tremendous increase in health care costs^2,7,8^, which will not be sustainable with the higher numbers of elderly persons forecasted in the coming decades^9^.

Osteoporosis is a multifactorial skeletal disease characterized by reduced bone mass and deterioration of the microarchitectural structure of bone tissue, with a resulting increase in bone fragility and fracture risk, and is a widely prevalent condition, in adults 50 years and older, and affecting twice as many women as men^10–12^. Fractures, which are widely prevalent complication of osteoporosis take a large economical toll on the individual, family, health care and society at large. This worrisome trend is predicted to continue. In the United States of America, the total annual direct and indirect expenditures for Medicare beneficiaries was approximately $57 billion in 2018 and is projected to increase to a staggering $95 billion by 2040^13^. In Europe, the total medical care costs for osteoporosis, including hospitalization and rehabilitation are also excessive: €37 billion in 2010^14^, with the corresponding projected costs for 2050 at €76.8 billion^15^. Besides personal and economic deficits, osteoporosis related fractures are a common cause for loss of personal independence and can pivot an individual with hip fracture from independence to dependent living^16,17^. It is vastly underappreciated that individuals with osteoporosis related fractures have a lower life expectancy^13,18,19^, plausibly due to fracture event, comorbidities or confounding musculoskeletal frailty that coexists with elderly individuals^20^. Indeed, 15% of Medicare beneficiaries experienced a second osteoporotic fracture, and 32% of beneficiaries died within two to three years of their first fracture. In addition, mortality rate instantly increases in the months of the initial fracture^21^.

Given these enormous estimates in terms of cost, quality of life and mortality, effective strategies to prevent and reduce the incidence of osteoporotic fractures must be swiftly implemented.

Fracture reduction strategies are complex, multi-dimensional and require recognition of ‘double whammy’ effect that drives the increased incidence of fragility fractures in the elderly, in whom the combination of two usually adverse circumstances- i.e., falls and underlying osteoporosis—frequently coexist together. This double association of increase fall frequency in presence of underlying osteoporosis is correlated with increased fracture incidence.

The current mainstay strategy to prevent fractures is to screen for osteoporosis by bone density test and then to treat individuals at high risk of fracture with anti-fracture pharmacotherapy. However, the strongest risk factor for fracture in a person with underlying osteoporosis is falls^22,23^. Despite this fact, assessment of fall risk is often overlooked as an important strategy to prevent fractures.

Postural balance is a primary independent risk factor for falls^24^. A previous study depicted that static and dynamic balancing ability in older women with osteoporosis significantly decreases as compared to an age-matched cohort, which increases fall risk in this group^25^. Wearable Inertial Measurement Unit (IMU) could be utilized to assess the physically frailty in fall prone individuals in variety of ways. Prior studies have determined that the dynamic test, such as gait speed has improved the possibility of forecasting fall prone individuals^26^. Many studies have discovered that slower walking speed was closely related with increased fall risk, and the IMU system is currently the most reliable system that can provide an accurate assessment of gait speed accurately^27–32^. The main cause of this phenomenon is the conscious compensatory gait mechanism, where fall prone people tend to intentionally adjust their gait speed to secure their steps. Reduced muscle mass, and strength as well as fear of falling were identified as mechanistic causes^33,34^. Moreover, static testing, such as postural sway is one of the most practiced assessment for fall risk^35–37^. Frames et al.^35^ reported that the obese faller has significant larger sway area and velocity compared to obese non-faller. Matinolli et al.^38^ has indicated that the Parkinson’s patients with falling experience has larger sway area compared to the non-fallers. Lastly, reduced physical activity level may indicate higher risk of fall^39^. Therefore, versatile application of the wearable system for accurately assessing these parameters would immensely support researchers and clinicians to prevent fall accidents, especially in individuals with osteoporosis who are more vulnerable to fractures^10,11^.

We hypothesized that gait characteristics that increase fall risk could be assessed in persons with osteoporosis with and without prior falls by using gait and postural stability parameters measured from a smartphone-wearable system. Additionally, we hypothesized that activity level (measured by inertial measurement unit [IMU]) would be different for persons who had falls than nonfallers. Given the close association between serum calcium, PTH, Vitamin D, albumin, and musculoskeletal health, we compared these markers in individuals with history of falls as compared to nonfallers.

## Results

Patient characteristics are reported in Table 1. Both groups were well-matched for age and body mass index. The mean (SD) age of the fall group was 75.6 (8.3) years and of the nonfall group 71.1 (9.7) years, 86% of participants were women (43/49). The mean (SD) body mass index was 24.9 (6.0) for the fall group and 23.5 (4.3) for the nonfall group.

**Table 1:** Characteristics of participants in the fall and nonfall groups (*p < 0.05, **p < 0.01, ***p < 0.001).

Characteristics	Mean (SD)		
	Fall group (n = 14)	Nonfall group (n = 35)	*P* value
Age, y	75.6 (8.3)	71.1 (9.7)	0.13
Women, No. %	11 (78.6)	31 (88.6)	
Men, No. %	3 (21.4)	4 (11.4)	
Height, cm	162.8 (8.0)	162.5 (9.8)	0.90
Weight, kg	65.2 (14.5)	62.36 (15.1)	0.55
BMI, kg/m^2^	24.9 (6.0)	23.5 (4.3)	0.35
Medications, No	5.57 (3.30)	3.50 (3.28)	0.05*
Total serum calcium, mg/dL (Reference range: 8.6–10.3 mg/dL)	9.37 (0.4)	9.67 (0.3)	0.01*
PTH, pg/mL (Reference range: 11–51 pg/mL)	79.14 (48.7)	40.23 (19.0)	0.002**
Albumin, g/dL (Reference range: 3.4–5.4 g/dL)	4.27 (0.33)	4.46 (0.28)	0.05*
Creatinine, mg/dL (Reference range: 0.6–1.3 mg/dL)	1.54 (2.2)	0.84 (0.21)	0.08
Vitamin D, ng/mL (Reference range: 25–80 ng/mL)	42.42 (15.34)	44.47 (13.60)	0.67
Dynamic physical activity level, %	8.36 (5.16)	17.56 (9.25)	0.002**
Sway area (cm^2^)	13.89 (14.90)	9.63 (11.04)	0.031*
Sway path length (cm)	36.17 (13.51)	30.69 (19.26)	0.053
Sway velocity (cm/s)	6.24 (2.33)	5.29 (3.32)	0.053
Dynamic stability, Lyapunov exponent (λ)	1.96 (0.21)	1.66 (0.08)	< 0.001***
Gait velocity (m/s)	0.79 (0.16)	0.96 (0.22)	0.012*

*BMI* body mass index, *PTH* parathyroid hormone.

We found no significant differences in sway path and velocity between the fall and nonfall groups but did find a significant difference in sway area (*P* = 0.031*). We also found significant differences in gait velocity (*P* = 0.012*) and dynamic stability (*P* < 0.001***) between the fall and nonfall groups. In general, participants in the nonfall group walked faster (0.96 m/s) than those who had fallen (0.79 m/s), and had better dynamic stability, as measured by the Lyapunov exponent (1.66). Furthermore, the nonfall group was much more active than the fall group at the 17.56% dynamic physical activity level as compared to 8.36% respectively (*P* = 0.002**).

Significant biochemical differences were noted in both groups. Participants in the fall group had a lower mean [SD] total serum calcium concentration (9.37 [0.4] mg/dL) than those in the nonfall group (9.67 [0.3] mg/dL) (*P* = 0.01*), higher parathyroid hormone (PTH) levels (79.14 [48.7] pg/mL) than the nonfall group (40.23 [19.0]) (*P* = 0.002**), and lower albumin levels (4.27 [0.33] g/dL) than the nonfall group (4.46 [0.28] g/dL) (*P* = 0.05*). Both groups had comparable serum vitamin-D and creatinine levels. Participants in the fall group took significantly more medications than those in the nonfall group. Furthermore, five deaths occurred over 3 years of the data collection effort (4 in the fall group [28.6%] and one in the nonfall group [2.9%]) (Table 1).

## Discussion

For older adults, walking, standing up from a chair, turning, and other activities are necessary for independent mobility. Gait speed, physical activities, and dynamic stability are independent predictors of the ability to perform ADLs as well as of the risk of falls and life expectancy^40^. In this study, we showed that persons with osteoporosis who had fallen within a year of entry into the study were less stable than those who had not fallen and exhibited unstable gait by dynamic gait pattern analysis (i.e., dynamic stability as measured by Lyapunov exponents). We also showed that individuals with osteoporosis at greater fall risk (due to occurrence of fall in prior year) could be differentiated using dynamic real-time measurements which can be easily captured by a smartphone app rather than by traditional postural sway and gait measures, which must be done in a laboratory setting.

A person’s inability to walk in a repetitive and stable manner predicts an evolving gait disorder that can lead to falls^41^. For those at the greatest risk for falling, the amount of variability during a linear gait analysis helps to quantify gait impairment. Furthermore, intracycle gait variability, despite no obvious gait impairment, may predict the potential for the gradual deterioration of stability mechanics. Thus, gait variability identified by nonlinear analysis could be a robust measure of a person’s neuromuscular function. Our finding calls for increased awareness of IMU device using a smartphone app as a simple and useful tool for evaluating and quantifying gait deficits of fall-prone individuals by providing important insights into the dynamic stability of walking.

Several other clinical and biochemical risk factors have been linked to a higher risk of falls in older adults with osteoporosis. Vitamin D and calcium are two nutrients essential for bone health. In our study, the fall group had lower serum calcium and higher PTH levels than the nonfall group. Vitamin D levels and kidney function did not differ between the two groups. Low serum calcium reflects a low dietary calcium intake or reduced intestinal calcium absorption and is one of several important causes of osteoporosis^42^. It also predicts significant muscle loss in adults^43^, thus calcium deficiency increases the risk of osteoporosis, sarcopenia and falls, serving as a catalyst for fractures. Similarly, Vitamin D deficiency causes lowering of bone density while lowering bone strength, thereby increasing instability, tendency to falls and fractures^44^. Vitamin D deficiency is corrected easily with over-the-counter supplements and is associated with better lower extremity function in older ambulatory adults, regardless of their physical activity or sedentariness^45^. Both low serum calcium and low Vitamin D results in secondary hyperparathyroidism, which when untreated contributes to bone loss, bone mineralization defects and ultimately increases incidence of hip and other fractures^46^. Elevated PTH^46^ and Vitamin D deficiency^47^ are also associated with muscle weakness. Elevated PTH levels are associated with significantly lower bone mineral density^48^ and have also been linked to falls independent of vitamin D level, especially in frail elderly persons. Studies conducted in nursing and assisted living facilities examined the association between serum PTH^49–52^ and falls and showed more falls among men and women with higher PTH levels (approximately 30% higher in one study)^49^. High PTH levels also significantly predicted time to first fall in another study of nursing and assisted living residents^50^.

Serum albumin is a biomarker of protein calorie malnutrition^53,54^, and low serum albumin is shown to be associated with frailty, leaving elderly individuals vulnerable to falls^55^. Our fall group had a significantly lower mean serum albumin level than the nonfall group. A low albumin level is closely related to future deterioration of appendicular skeletal muscle mass in older adults, which can lead to sarcopenia^56^. A lower serum albumin level has been cross-sectionally related to the decline of muscle force; after three years, the muscle intensity of persons in a longitudinal study decreased significantly^57^.

Polypharmacy exposure increases the risks of numerous negative health consequences for elderly persons, including falls^58–60^. Our study supports this association; those in our fall group used significantly more medications than those in the nonfall group.

Data from US National Vital Statistics System mortality files show an increase in mortality from falls particularly with advancing age^61^. Our data is concordant with these results. In our study, the all-cause mortality was 28.6% (4/14) for those with falls versus 2.9% (1/35) for those without.

### Strengths and limitations

Strength of our study is as follows; our study data were obtained from a community-based clinic in an ambulatory setting reflecting real world situation. Standard methods were used for all assessments and data collection. Furthermore, 3-day assessments of ADLs were done with the participants wearing a portable IMU system and recording activities manually in a journal, which allowed researchers to make exact correlations. Our study has following limitations, the study was done in open-label fashion; thus, participants were aware that gait was being measured. From the gait assessment, we only focused on the gait speed, which is most fundamental data for fall risk and depicts the overall frailty status. Osteoporosis is more prevalent in women, thus as anticipated significantly more women (86% of participants) participated in the study, results of our study may not be applicable to men. It should be noted that hypothesis of this study was not focused on gender differences on fall mechanisms but focused on fall and nonfall groups regardless of their gender did not evaluate dietary calcium intake or calcium supplementation. Our study had a small number of participants. Finally, we did not adjudicate the cause of death in the groups.

## Methods

### Participants

To be included in the fall group, participants had to have fallen once in the year before they entered the study. To be included in the nonfall group, participants could have no falls within the year previous to study entry. We included adult men and women over the age of 50 years with a diagnosis of osteoporosis (with and without prior fragility fracture) who were living and ambulating independently. We excluded patients with a history of fractures not due to osteoporosis (such as pathologic fractures due to cancer metastases) and major comorbid conditions (such as dementia or visual problems). A research affiliate (S.M) following the participant recruitment protocol, asked eligible patients whether they were interested in being part of the study. If the patient agreed to participate, a physician (K.B.D., M.D.W.) discussed the study with the patient, answered all relevant questions. Participants were enrolled after written informed consent. The research was approved by the Mayo Clinic IRB (and Arizona State University IRB). All research was performed in accordance with relevant guidelines and regulations.

### Instrumentation

A smartphone (with inbuilt IMU) with a holster and clip was used for monitoring. The IMU sensor signals were sampled at 50 Hz by using the customized smartphone app Lockhart Monitor^62^ (Locomotion Research Laboratory, Arizona State University, available through the iOS App Store), and longitudinal data were collected by using the DynaPort MoveMonitor+ IMU device (Motion Monitor+ , McRoberts BV, The Hague, Netherlands) at 100-Hz frequency. The Lockhart Monitor has the capability of assessing linear and nonlinear parameters of a person’s gait and postural stability. Further data processing was accomplished using custom-made MATLAB routines (MATLAB version 9.3, 2017, The MathWorks Inc). The mobile app consists of a start and stop button and recorded voice instruction, with ample rest duration built in between each performed activity. The signals were truncated using the temporal information of voice commands through the app.

### In-clinic data collection and analyses

**Figure 1:**
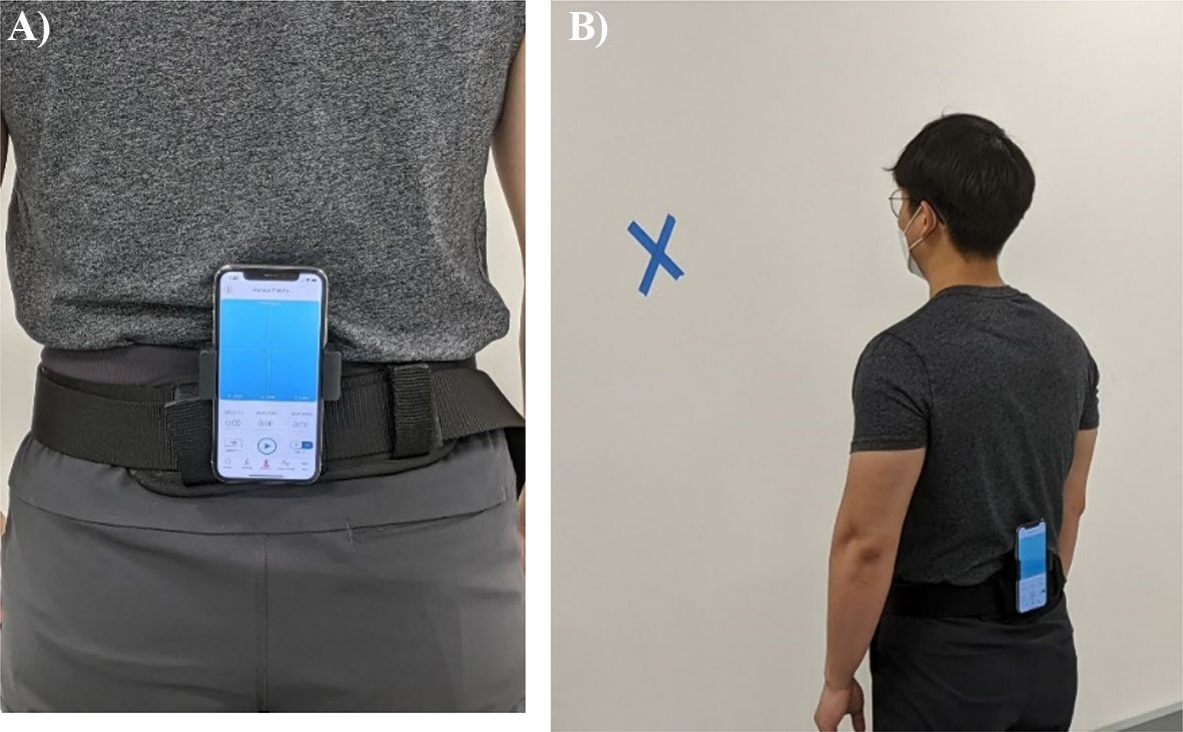
(**A**) A smartphone was affixed in the participant’s lumbar region for the in-clinic walking speed and postural stability assessments. (**B**) All participants were required to perform postural stability testing for 60 s with eyes open and closed. The cross on the wall provided a visual cue for the participants.

Participants' blood samples were collected by a licensed phlebotomist at the study site or at a CLIA-certified laboratory (2 × 10 mL whole blood). Various standardized biochemicals were extracted and reported. For the testing procedure (Fig. 1), participants were asked to maintain their natural standing posture for 60 s in 2 different situations: eyes open and eyes closed for 2 times each. For the gait speed assessment using the 10-m walking protocol, the smartphone data collection was begun at the initial footfall after the start line and automatically stopped with the first footfall after the 10-m line. This automated assessment was determined by the threshold algorithm, which is a sum of mean and two standard deviation of the variance from the 5 s of fixed standing calibration session^63^. This process was repeated twice with adequate time for the participants to recuperate between trials. The walking speed and other linear gait parameters were securely saved within the IMU system embedded in the smartphone for later processing.

In the clinical environment, we measured participants’ postural stability (or postural sway) and their walking velocity (ie, gait velocity or walking speed)^36,64,65^. To analyze the sway area from the postural stability, mean sway radius was calculated with anterior/posterior and medial/lateral movement of center of mass divided by the sample of data points (*n*) and multiplied by pi (π). Sway path length was computed with the summation of Euclidean distance among the points during the total sway period. Sway velocity was calculated with sway path length divided by the total sway period.

1 $${\text{Sway Area }}\left( {{\text{cm}}^{{2}} } \right) = \left( {\frac{{\sqrt {x^{2} + y^{2} } }}{n}} \right)^{2} {*}\pi$$

2 $${\text{Sway Path Length }}\left( {{\text{cm}}} \right) = \mathop \sum \limits_{n - 1}^{n} \sqrt {(x_{n} - x_{n - 1} )^{2} { } + { }({\text{y}}_{n} - {\text{y}}_{n - 1} )^{2} { }}$$

**Figure 2:**
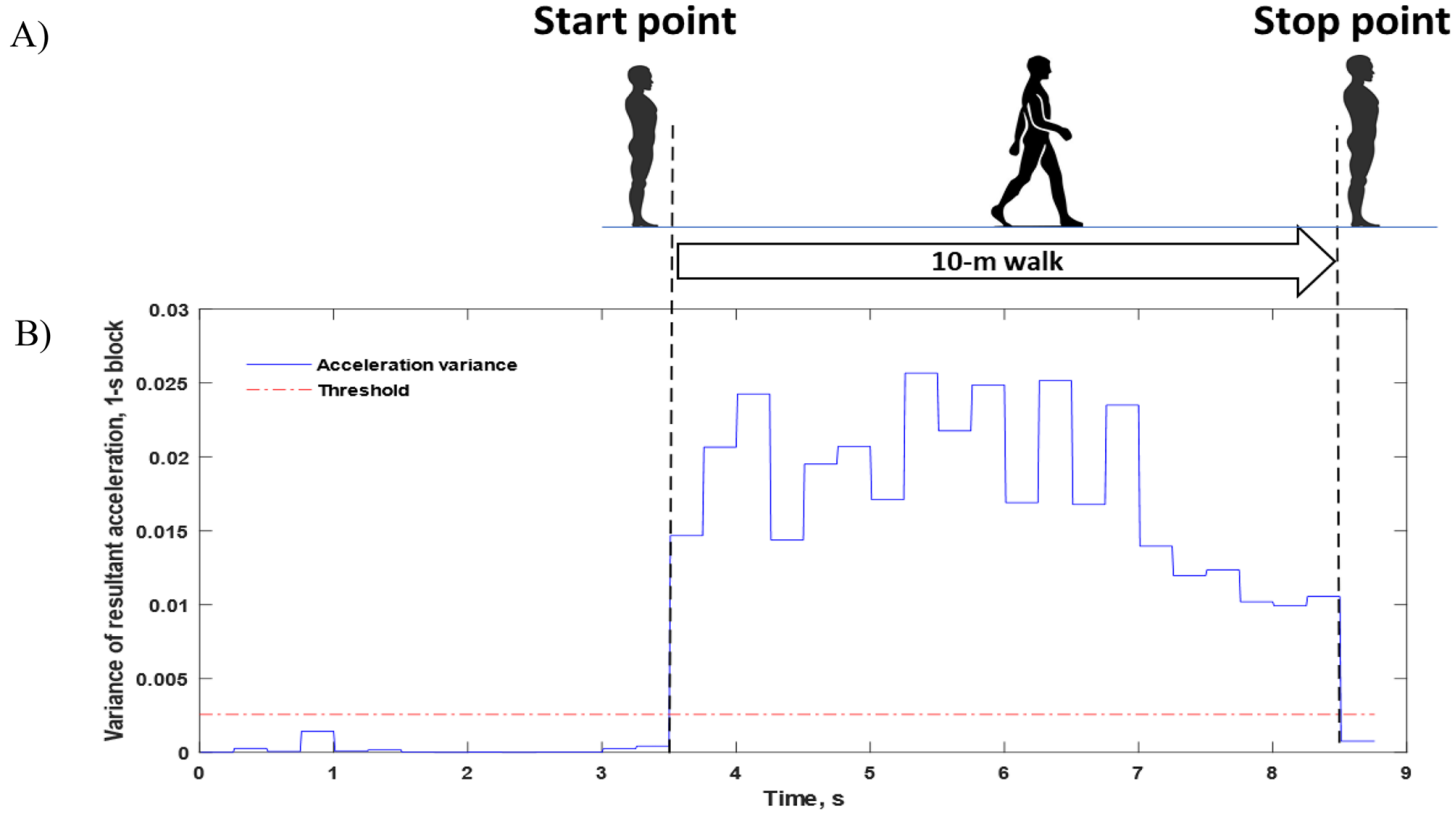
Ten-Meter Walking Speed Protocol and Gait Analysis. (**A**) Gait speed assessment is initiated automatically as the participant takes a step from standing still. After the participant steps completely over the 10-m marker and stands still again, the assessment is completed. (**B**) Acceleration signals the moving window (0.5 s) variance of low-pass-filtered resultant acceleration, which was used to calculate the gait speed.

Figure 2 illustrates the 10-m walking speed protocol and the assessment and analysis of the ambulatory signal from the IMU. To compute the gait velocity from this acceleration data, the total distance (*d*) was 10 m, and it was divided by the period of the time (*t*) that participant took to complete the entire walking distance.

3 $${\text{Gait velocity }}\left( {{\text{m}}/{\text{s}}} \right) = \frac{d}{t}$$

For the dynamic stability assessment (i.e., the nonlinear dynamic measure of the short term Lyapunov Exponent (LyE)^41,66^), a 3-min continuous walking exercise was performed on a clear uncluttered pathway at Mayo Clinic. For this assessment, participants were asked to walk continuously for 3 min at their normal walking speed while wearing a smartphone at their sacral area. To calculate the LyE, time-delayed coordinate method was applied. This method indicates that any adequate size of fundamental dynamic information that is performed in single dimension temporal time series can be reconstructed into multi-dimensional state spaces. After determining the state space, all the nearest neighbors were collected which has the closest distance from the trajectories^67^.

### Longitudinal data collection and analyses

Longitudinal data collection was conducted at the participants’ dwellings. Participants were asked to maintain an activity journal reflecting their activities of daily living (ADLs). Activities during the day were categorized with four main movements such as sitting, standing, walking, and lying down. Participants were instructed to log in these motions on a minute scale, to ensure that activities were recorded accurately (Table 2). They also reported the location where activity was performed, described the activity as well as the type of movement required. In the non-clinic environment, participants’ activity levels were measured as the percent average each day.

**Table 2:** Example of activities of daily living journal from a participant.

Time (h:min:sec)	Activity	Duration, min	Location	Comment
12:30:00	Walking	5	Clinic	Floor
12:35:00	Sitting	7	Clinic	Chair
12:42:00	Walking	1	Clinic	Floor
12:43:00	Sitting	9	Car	Chair
13:56:00	Laydown	4	Home	Bed
14:00:00	Laydown	136	Home	Bed
16:16:00	Walking	24	Home	Floor
16:40:00	Standing	4	Kitchen	Floor
16:44:00	Sitting	3	Home	Chair
16:47:00	Walking	3	Home	Floor

**Figure 3:**
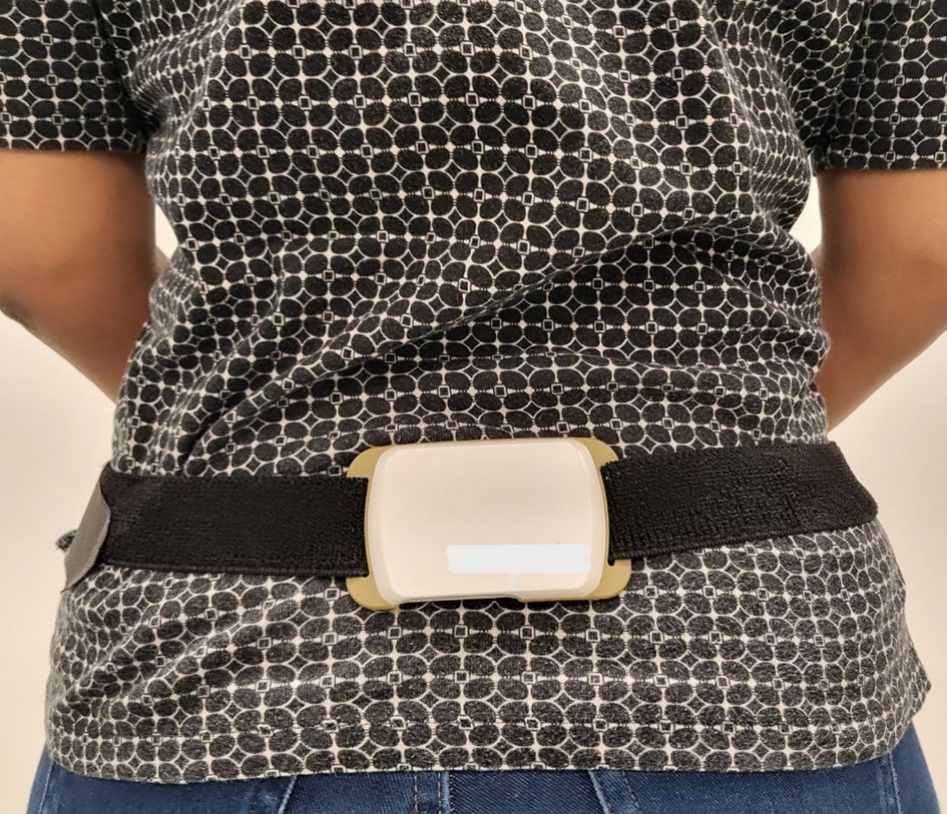
DynaPort MM + IMU device is affixed on the participant’s sacrum region to perform 3 days of Activities of Daily Living data collection.

**Figure 4:**
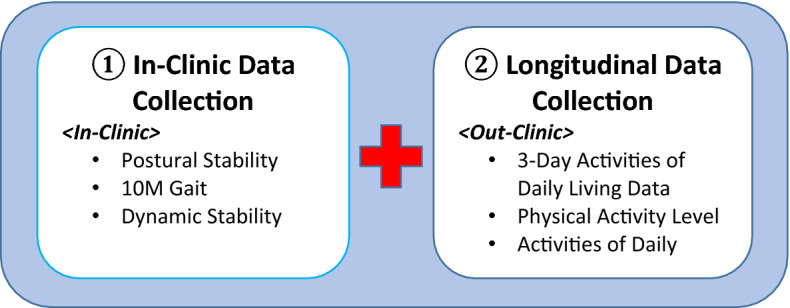
Study Design Layout for the 1. In-clinic Data collection, 2. Longitudinal Data Collection.

ADLs data was also collected for 72 h via the DynaPort MM + IMU device located at the sacral part of the spine (Fig. 3). Activity journal was independently reviewed (by S.M.) to ensure concordance with the DynaPort data. Participants were allowed to disconnect the sensor only when bathing or swimming. Longitudinal data were analyzed with MATLAB. The X, Y, and Z coordinate acceleration data were refined with high- and low-pass Butterworth filters to remove noise from the raw data. Subsequently, the 1-Hz cut-off frequency was modified to determine the dynamic physical activity level of the participants^68–70^. This algorithm allowed us to compare ADL activity levels between participants with and without falls. Figure 4 summarizes the procedure for in-clinic and 3-day longitudinal data collection.

### Statistical analyses

Dependent variables were analyzed using multivariate analysis of variance (MANOVA). Wilk Λ test was used to determine which factors of MANOVA were most relevant to participants in the fall versus nonfall groups. Then, univariate analyses (1-way analysis of variance) were performed on each of the dependent variables with each participant treated as a random variable, using falling versus nonfalling as significant factor (α = 0.05) (Table 1).

## Data Availability

The datasets are not publicly available due to restrictions used under the license for the current study. There are available on reasonable request from the corresponding author.
